# Reducing Interface Defects and Porosity of Adhesive Bonded Aluminum Alloy Joints via Ultrasonic Vibration

**DOI:** 10.3390/polym15092098

**Published:** 2023-04-28

**Authors:** Hui Wang, Guodong Kang, Yizhe Chen, Zhaoyi Liu, Lin Hua

**Affiliations:** 1 Hubei Key Laboratory of Advanced Technology for Automotive Components, College of Automotive Engineering, Wuhan University of Technology, Wuhan 430070, China; 2 Hubei Longzhong Laboratory, Xiangyang 441000, China; 3 Hubei Collaborative Innovation Center for Automotive Components Technology, Wuhan 430070, China; 4 Hubei Research Center for New Energy & Intelligent Connected Vehicle, Wuhan University of Technology, Wuhan 430070, China

**Keywords:** sandblasting, adhesive bonding, ultrasonic vibration, interface defect, cavitation

## Abstract

The surface microstructure formed by physical or chemical modification is essential for the desired joint strength. However, defects in the bonding interface and adhesive can be found. Such defects decrease shear strength and durability. In this study, ultrasonic vibration was applied to liquid adhesive on the sandblasted aluminum alloy plates. With ultrasonic treatment, the joints obtained the compact bonding interfaces and lower porosity of the adhesive layer. The treatment improved the shear strength by 9.1%. After two weeks of hydrothermal aging, the shear strength of joints only sandblasted decreased drastically by 48.9%, while it was 14% for the joints with ultrasonic vibration. The cavitation effect in the adhesive was detected by the aluminum foil erosion method. The result showed that a great number of micro-jets generated by the cavitation effect have intensive impact on the bonding interface which provide the adhesive with powerful force to fill the micro-grooves. Another finding in this work is that bubbles were gathered in the adhesive away from the vibration area. This mechanism was successfully used to reduce the porosity of the adhesive layer of joints.

## 1. Introduction

In the automobile and aerospace industries, the connection of multiple materials has become widespread and vital [1,2]. Compared with mechanical fastening and welding, the benefits of adhesive bonding are even stress distribution [3], good fatigue [4] and impact performance [5], the prevention of galvanic corrosion, and weight reduction [6]. However, its insufficient durability in extreme environments limits its application in certain circumstances [7,8].

In order to achieve the desired bonding strength, it is often necessary to modify the bonding surface physically or chemically. Physical treatment methods include mechanical grinding [9], sandblasting [10], and laser ablation [11], which can not only remove the contaminants on the bonding surface, but also increase the surface roughness. Chemical methods include anodic oxidation [12], silane treatment [13], and plasma treatment [14]. After anodizing treatment with sulfuric acid, a porous oxide film was formed on the surface of the aluminum alloy. This porous structure allows the adhesive to penetrate into the nanopores to form mechanical interlocking. Silane and plasma treatment, however, have little effect on the surface morphology. Their combination with physical treatments can further improve the bonding strength [15].

A rough surface improves the bonding strength through an extended bonding area and the mechanical anchoring effect [16]. It also presents a challenge for paste adhesive to completely wet the interface because of its high viscosity and low fluidity [17,18]. The defects in the interface limit the bonding strength and affect the joints’ environmental durability. One of the most critical factors affecting the durability of bonded joints is moisture [19], which has reversible [20,21] and irreversible [22] influences on mechanical properties and durability. For bonded joints, interface defects allow a faster moisture diffusion at the interface than in the adhesive layer owing to capillary adsorption [23]. The locus of joint failure was changed from cohesive in the adhesive to along the bonding interface due to the corrosion caused by moisture [24].

The ultrasonic wave has unique effects in cleaning technology [25], the homogenization and dispersion of multi-component systems [26], degassing, and chemical synthesis [27]. Recently, some researchers have found that ultrasonic vibration is significant in the process of bonding. Wang et al. [28] presented an approach to characterize the capillary effect of a paste adhesive. They found a greater rise in the adhesive in a capillary tube when ultrasonic vibration was applied. Du et al. [29] found polypropylene flowed into the aluminum alloy surface’s porous structure via an ultrasound-assisted hot-pressing technology. Holtmannspotter et al. [30] utilized ultrasonic cavitation to remove the contaminants on the adherend to minimize their influence on bonding strength. Ultrasonic vibration has been widely used in promoting the filling and wetting of adhesive to strengthen its connection capacity with other materials. However, less relevant research has focused on the other effects of ultrasonic vibration in the adhesive and their contribution to the joints.

In this work, sandblasting was chosen to modify the surface morphology of the aluminum alloy plates. Before the adhesive was cured, 20 kHz ultrasonic vibration was applied to the adhesive to reduce the defects in the interfaces of joints. An orthogonal experiment was conducted to optimize ultrasonic parameters. The hydrothermal aging process was conducted to evaluate the durability of the joints. The joints’ shear strength, failure mode, and interface morphology were investigated. The aluminum foil method was used to detect and analyze the ultrasonic cavitation effect in the adhesive.

## 2. Materials and Methods

### 2.1. Materials

In this work, 7075 aluminum alloy plates (Dongguan Gaoduanda Co., Ltd., Dongguan, China) were used as the adherend, with a tensile strength of approximately 560 MPa. The chemical composition is listed in Table 1.

A dual-component adhesive LOCTITE EA 9309.3NA was used to join the aluminum alloy plates. According to the product manual, the cured adhesive properties are listed in Table 2.

The adhesive components A and B were mixed at a mass ratio of 100:22 at room temperature. It was appropriate to manually mix the adhesive with a glass rod owing to the small amount of adhesive (less than 10 g) used at a time. The pot life (450 g mass) of the mixed adhesive was approximately 35 min at 25 °C. The adhesive could be cured for 3 to 5 days at 25 °C or 1 h at 82 °C.

### 2.2. Pretreatment of Aluminum Alloy Plates

**Figure 1:**
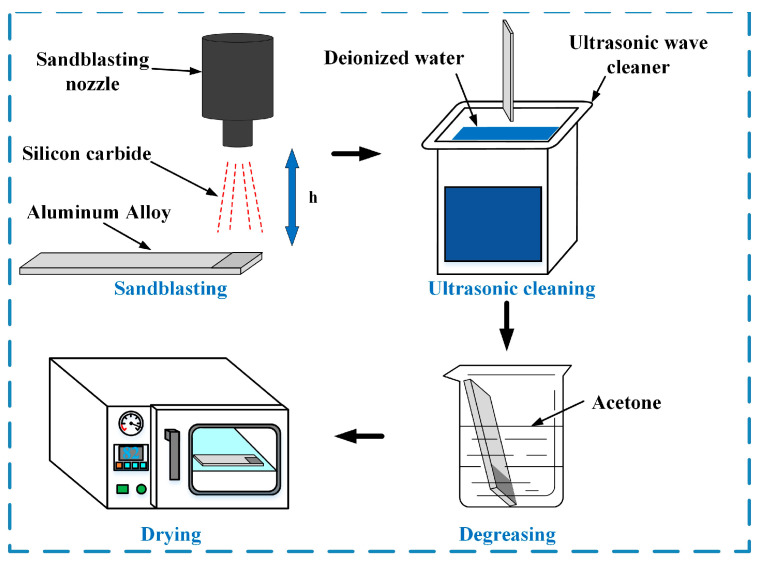
The pretreatment process of aluminum alloy plates.

The pretreatment process of the aluminum alloy plates is shown in Figure 1. The first step was sandblasting. The sandblasting device WLX-1010 (Wuhan Weilixin Machinery Equipment Co., Ltd., Wuhan, China) was used. According to the optimization of sandblasting parameters, all plates were sandblasted with 80-mesh silicon carbide under a pressure of 0.5 MPa. During sandblasting, aluminum alloy plates were placed 10 cm below the silicon carbide outlet, and the sandblasting time was 4 s for one plate. This method could remove the surface oxide layer and form microstructures on the surface. The sandblasted plates were cleaned with deionized water using a 40 kHz ultrasonic wave cleaner JP-010S (Skymen Cleaning Equipment Shenzhen Co., Ltd., Shenzhen, China). It has a 2 L capacity and 60 W ultrasonic power (1 acoustic source). In order to ensure the cleaning effect of the plates, only 10 plates were cleaned at a time for 5 min at room temperature. After that, the plates were degreased by placing them in a beaker containing acetone. This process will be completed in 1 min at room temperature. Then, the plates were wiped with dry non-woven fabric and placed in a vacuum-drying oven at 82 °C for 3 min to remove the residual acetone on the surface. The vacuum-drying oven DZF-6020A (Shanghai Kuntian Laboratory Instruments Co., Ltd., Shanghai, China) was used. It has 1.2 kW power and ±1 °C temperature fluctuation (from 50–250 °C).

### 2.3. Preparation of Single-Lap Joint

**Figure 2:**
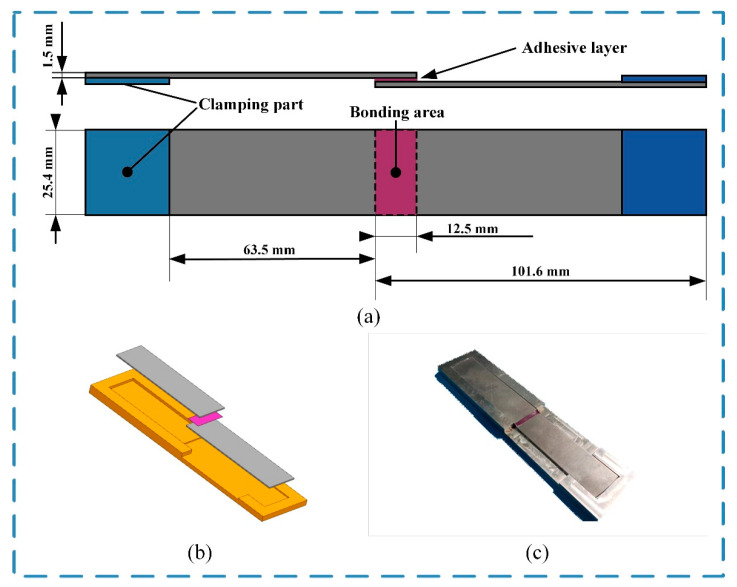
The single-lap joint: (**a**) the size of the joint (unit: mm), (**b**) the fixture, and (**c**) the joint assembly.

The size of the joint was designed according to ASTM D1002 [31]. The single-lap joint is shown in Figure 2a. The aluminum alloy plate size was 101.6 × 25.4 × 1.5 mm^3^, and that of the adhesive layer was 25.4 × 12.5 × 0.2 mm^3^.

In order to ensure consistency in the size of the adhesive layer, the joints were prepared using a fixture made of 7075 aluminum alloy. The fixture was designed to have two cavities with a height difference of 1.7 ± 0.02 mm. The upper cavity was 89.1 × 25.4 mm^2^, and the lower was 101.6 × 25.4 mm^3^, as shown in Figure 2b. The adhesive will be prepared when the aluminum alloy plates and fixtures are ready. There was no extra interval before applying the adhesive to the bonding surface of the aluminum alloy plates. Only 5 joints were made at a time, so the adhesive was only applied to the bonding surface of 10 plates. This process will be completed in 2 min manually with a spatula and has less influence on the adhesive pot life. Two of the plates applied with adhesive were overlapped in the fixture. After the excess adhesive was removed, the fixture with the uncured joint was placed in a vacuum-drying oven for curing, in which a curing period of 1 h at 82 °C was selected with atmospheric pressure.

### 2.4. Ultrasonic Vibration Treatment

**Figure 3:**
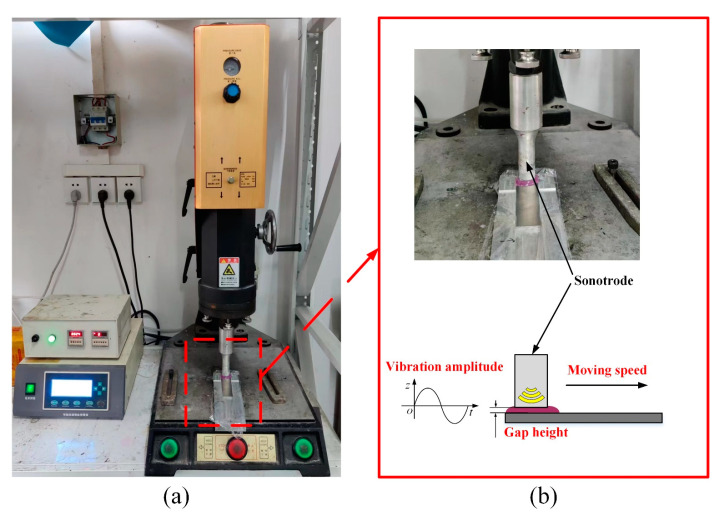
(**a**) Experimental platform of ultrasonic process, and (**b**) its schematic.

Ultrasonic vibration treatment was applied to the adhesive after it was applied to the aluminum alloy plate, as shown in Figure 3. Figure 3a shows the ultrasonic vibration equipment ME-1800 produced by Maxwide Ultrasonic Co., Ltd., Shanghai, China. The ultrasonic transducer converted the high-frequency AC electrical signals into 20 kHz ultrasonic vibration. After being amplified by the horn, the ultrasonic vibration was transmitted to the sonotrode. Finally, the ultrasonic vibration was exerted on the adhesive when the sonotrode, driven by a pneumatic cylinder, came into contact with the adhesive, as shown in Figure 3b. There are three key parameters of the ultrasonic vibration treatment: the vibration amplitude, the moving speed of the sonotrode in the adhesive, and the gap height between the sonotrode and aluminum alloy plates. These parameters were optimized by an orthogonal experiment design, including 24 μm amplitude, 4 mm/s moving speed, and 1.5 mm gap height. The orthogonal experiment design will be presented in detail in Section 3.1. After the ultrasonic vibration treatment, the plates with adhesive were overlapped in the fixture, and then were thermally cured in the vacuum-drying oven.

### 2.5. Shear Strength Test

A universal tensile testing machine (SANS CMT5205 manufactured by MTS Systems (China) Co., Ltd., Beijing, China) was used to measure the maximum failure load. The device has a 200 kN maximum rated force capacity and ±0.5% force accuracy. According to the standard ASTM D1002-10 [31], two heel blocks of 1.7 mm thickness were placed at both ends of the plates to eliminate the influence of the bending moment. The tensile tests were carried out at a constant speed of 2 mm/min. The shear strength was calculated by the following formula:

(1) $$\tau =\frac{F}{S}$$

where *F* represents the maximum failure load (N) of the bonded joint, *S* represents the bonding area (m^2^), and *τ* represents the shear strength (MPa).

### 2.6. Hydrothermal Aging Process

A hydrothermal aging process is used to test the durability of the bonded joints in a humid environment. The experimental conditions are listed in Table 3. The cured joints were put into a beaker with 5% NaCl solution. Then, they were all placed in the vacuum- drying oven at 80 °C for two weeks. After the hydrothermal aging process, the joints were dried at 80 °C for 10 min before testing their shear strength.

### 2.7. Experimental Plan

In order to evaluate the improvement in the bonding performance of aluminum alloy joints by the application of ultrasonic vibration, five groups of experiment were designed, as shown in Table 4. The adopted surface treatments including sandblasting, ultrasonic cleaning, and degreasing in the plan are described in Section 2.2. The difference between each group of experiments lies in whether there is ultrasonic vibration treatment in the bonding process of aluminum alloy plates and whether there is a hydrothermal aging process before the shear strength test. Group A was a reference experiment where the aluminum alloy plates were only cleaned and degreased before bonding, without sandblasting. Among the other four groups, the ultrasonic vibration treatment with optimized parameters (detailed in Section 2.4) was only included in the bonding process of joints in Group D and Group E. When the joints were cured, the joints in Group B and Group D were directly subjected to the shear strength test, while the joints in Group C and Group E were tested after experiencing the hydrothermal aging process.

### 2.8. Morphology of Interface

In order to characterize the defects inside the bonded joints, the interface of aluminum alloy/adhesive was observed by scanning electron microscope (SEM) Tescan MIRA4 (Tescan, Shanghai, China). Using a water cutter LTJ1613-5A (Shanghai Lionste Co., Ltd., Shanghai, China), the cured joints were cut into samples of 10 × 10 × 3.4 mm^3^. The equipment has ±0.04 mm/m positioning accuracy, ±0.1 mm/m kinematic accuracy, and 8 m/min maximum cutting speed (X and Y axes). The joints were cut by the mixture of water and 80-mesh silicon carbide with 380 MPa pressure, 3.8 L/min flow rate, and 3 m/min cutting speed. Then, the samples’ cross-sections were polished by 400-mesh, 1000-mesh, 2000-mesh, and 4000-mesh sandpapers and polishing cloth sequentially. After the samples were cleaned and coated with platinum, their cross-sections were observed by SEM with accelerating voltages of 5 kV under a magnification of 500×. The results were used to analyze the effect of the ultrasonic vibration on the bonding interface.

### 2.9. Ultrasonic Cavitation Experiment

**Figure 4:**
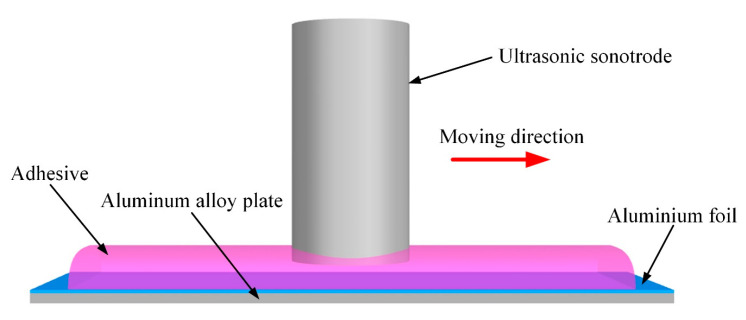
Ultrasonic cavitation experiment.

The aluminum foil method was used to detect cavitation in the adhesive. The micro-jets generated by ultrasonic cavitation can impact the nearby solid surface, a process called cavitation erosion. The action of the micro-jets can be detected more obviously by using soft materials, in which aluminum foil is recommended. In this work, the 50 μm thick aluminum foil was pasted onto the aluminum alloy plate to detect the cavitation effect in the adhesive. The schematic of this experiment is shown in Figure 4. The experiment was conducted at room temperature. When the prepared adhesive was applied onto the surface of aluminum foil, the ultrasonic sonotrode was controlled to enter and exert ultrasonic vibration on the adhesive. The parameters include 20 kHz frequency, 24 μm amplitude, 1.5 mm gap height, and 4 mm/s moving speed, which were values consistent with the treatment adopted in manufacturing single-lap joints. After the ultrasonic vibration treatment, the adhesive on the aluminum foil was cleaned with acetone. Using Zeiss Axio Scope.A1 (Beijing Precise Instrument Co., Ltd., Beijing, China), the aluminum foil surface was illuminated by the adjusted halogen lamp HAL 100 and captured under a magnification of 200×.

## 3. Results

### 3.1. Orthogonal Experimental Design

The orthogonal experimental design was used to study the multi-factors and multi-levels of the ultrasonic vibration treatment. The joints were subjected to different schemas of ultrasonic vibration to obtain optimal parameters for the adhesive bonding. According to the trial experiments, the parameters mainly included the gap height, moving speed, and vibration amplitude. The gap height is the distance between the end face of the sonotrode and the surface of the aluminum alloy plate. The moving speed is the relative speed of the sonotrode in the adhesive. When the ultrasonic sonotrode was controlled to enter the adhesive, its horizontal movement was determined by moving the aluminum alloy plates. A slower speed means a longer vibration time for the adhesive. The vibration amplitude is the maximum vibration displacement of the sonotrode. The factors and the four levels of orthogonal experiment are listed in Table 5.

**Figure 5:**
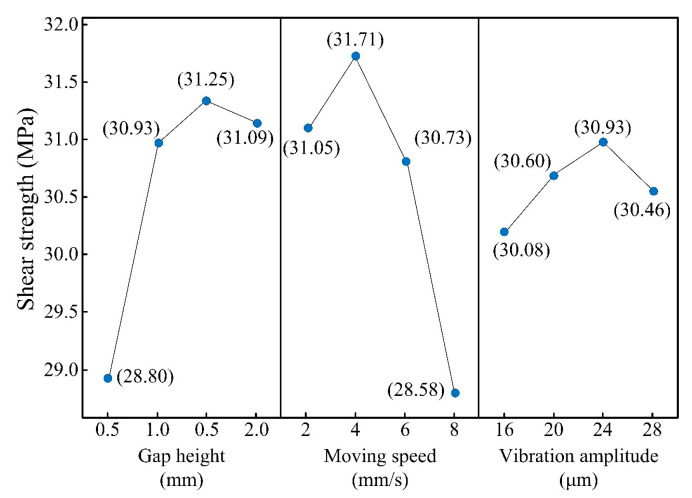
Main effect plot of means.

Based on Table 5, the orthogonal experiment was designed using the Minitab software (V19). The experimental schemes are listed in Table 6. In order to reduce experimental error, the order of the schemes was randomly determined by drawing lots. Each scheme of the orthogonal experiment was repeated 5 times and for 80 joints in total. After the shear strength test, the maximum failure load and the actual bonding area were obtained. The shear strength was calculated according to the formula introduced in Section 2.5. The mean value and standard deviation of 5 joints in each scheme are shown in Table 6. Then, the results were analyzed using “Taguchi Design Analysis” in Minitab. The mean response of each factor and level is shown in Table 7, and the main effects are given in Figure 5.

The mean response is the average value for all the schemes of the specified factor level in Table 6. From Table 7, the influence rank of the factors in descending order is moving speed > gap height > vibration amplitude. The main effect refers to differences among level means for one or more factors, which are present if different levels of a factor affect the response differently. Figure 5 shows the trend of the change in shear strength with factor levels. It shows that the gap height of 1.5 mm, the moving speed of 4 mm/s, and the vibration amplitude of 24 μm are the optimal parameters for the ultrasonic vibration treatment. As the optimal combination was not included in the orthogonal experimental schemes, additional experiments were conducted for verification. Table 8 (Group D) shows the results using the optimal scheme. The average shear strength (33.33 MPa) was higher than that shown in Table 6, which proved its validity.

### 3.2. Shear Strength Testing Results

**Figure 6:**
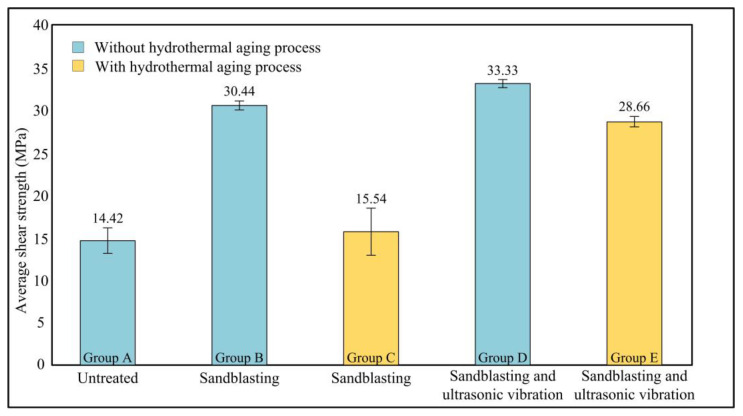
The average shear strength of the five groups of joints.

As mentioned in Section 2.5, five groups of joints were designed to characterize the effects of the ultrasonic vibration on the adhesive bonding. Each group was repeated five times and for 25 joints in total. The shear strength test results are shown in Figure 6.

The average shear strength of the joints in Group A was 14.42 MPa. For Group B and Group D, where the joints did not experience hydrothermal aging, the ultrasonic vibration treatment increased the strength from 30.44 MPa to 33.33 MPa. However, after the hydrothermal aging process, the shear strength decreased by different degrees. The strength of the joints in Group C was only 51.1% that of Group B, although the joints were bonded similarly. As for joints treated by ultrasonic vibration, the strength of Group E was approximately 86% that of Group B. The above dates illustrate that ultrasonic vibration can improve the bonding performance of aluminum alloy joints, especially in a humid environment.

**Figure 7:**
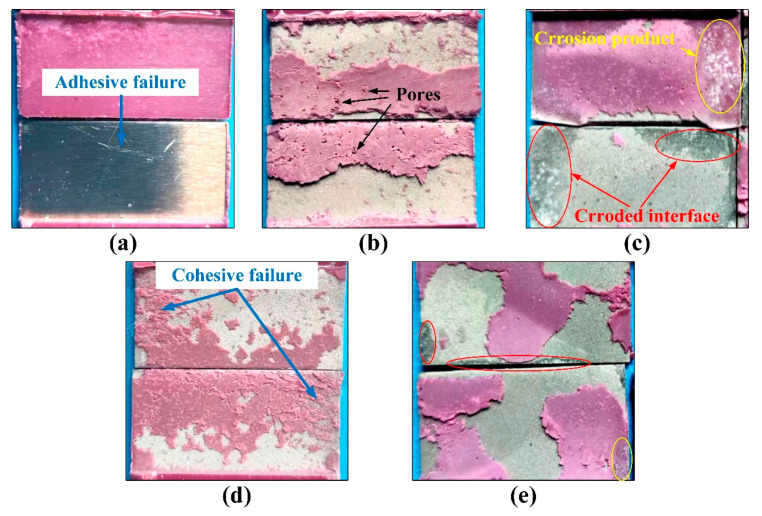
Failure surfaces of (**a**) Group A, (**b**) Group B, (**c**) Group C, (**d**) Group D, and (**e**) Group E.

The joint failure surfaces after the shear strength test are shown in Figure 7. Their failure modes were analyzed according to the standard ASTM D5573-99 [32]. For the joints in Group A, the failure occurred at one bonding interface, representing adhesive failure. It indicates poor bonding performance of the original aluminum alloy surface. After sandblasting, the joints in Group B showed a mixed failure mode of adhesive failure and cohesive failure. It indicated that the sandblasting treatment could markedly improve the bonding performance of the aluminum alloy plate surface. After hydrothermal aging, however, the joints in Group C represented interface failure, as shown in Figure 7c.

Additionally, severe corrosion was found on the bonding interface in Group C. It indicated that the external moisture undermined the bonding interface. With the ultrasonic vibration treatment, the joints in Group D were classified as the mixed failure mode, although the same as Group B. The torn area of the adhesive was more evident, and fewer adhesive pores were found, indicating that the ultrasonic vibration treatment could further strengthen the adhesion of the sandblasted plate. The joints in Group E underwent the same hydrothermal aging process as Group C. It can be seen that only slight corrosion occurred at the edge of the bonding area, proving that the ultrasonic vibration treatment could effectively protect the joint’s bonding interface from external moisture.

### 3.3. Interface Morphology Analysis

**Figure 8:**
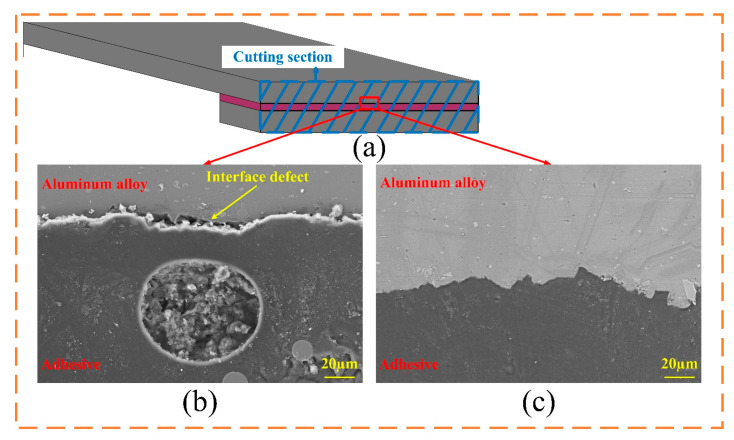
Cross-section of the joints: (**a**) schematic, (**b**) without ultrasonic vibration treatment, and (**c**) with ultrasonic vibration treatment.

The joints from Group B and Group D were made into a sample to observe their bonding interfaces. Under a magnification of 500×, it can be seen that the adhesive was not in contact with the sandblasted surface completely, as shown in Figure 8b. In addition to decreasing the shear strength, those defects could form the capillary paths for external moisture when the joints are exposed to a humid environment, which corroded the bonding interfaces, as shown in Figure 7c. The sample shown in Figure 8c was treated by ultrasonic vibration. The adhesive was in good contact with the adherend. A more compact bonding interface can enlarge the contact area and strengthen mechanical anchoring between adhesive and adherend. Thus, achieving the higher shear strength of the joint. Moreover, reducing the interface defects can efficiently cut off those capillary paths, which could explain only slight corrosion found on the edge of the bonding area.

### 3.4. Ultrasonic Strengthening Analysis

**Figure 9:**
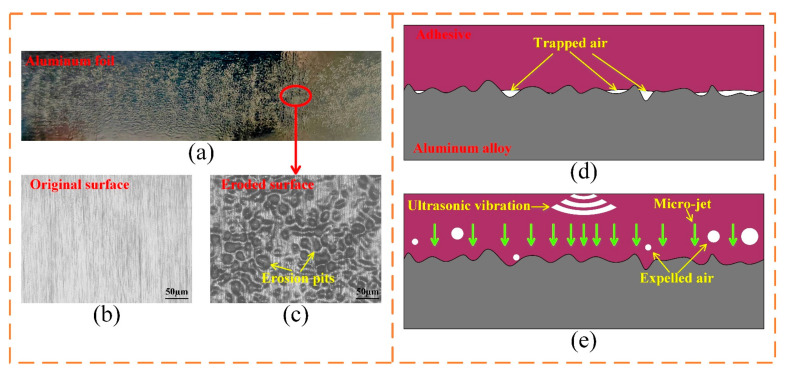
Cavitation effect in the adhesive: (**a**) tested aluminum foil, (**b**) original surface, and (**c**) erosion pits under magnification, and illustrations of bonding interface without (**d**), and (**e**) with the ultrasonic vibration treatment.

Cavitation includes shock wave, micro-jet, and luminescence. The aluminum foil erosion method (detailed in Section 2.9) was used to detect the cavitation in the adhesive by the action of micro-jets to soft aluminum foil. The result is shown in Figure 9.

In the cavitation experiment, the optimal ultrasonic scheme was adopted. The treated aluminum foil is shown in Figure 9a. The detailed characteristics of the aluminum foil are shown in Figure 9b,c. A great number of micro-jets were generated in the adhesive and impacted on the surface of the adherend. The erosion pits on the surface of aluminum foil proved that the cavitation effect has intensive impact on the bonding interface than other effects of ultrasonic vibration in the adhesive, which resulted in more contact between the adhesive and micro-grooves, as shown in Figure 9d,e.

**Figure 10:**
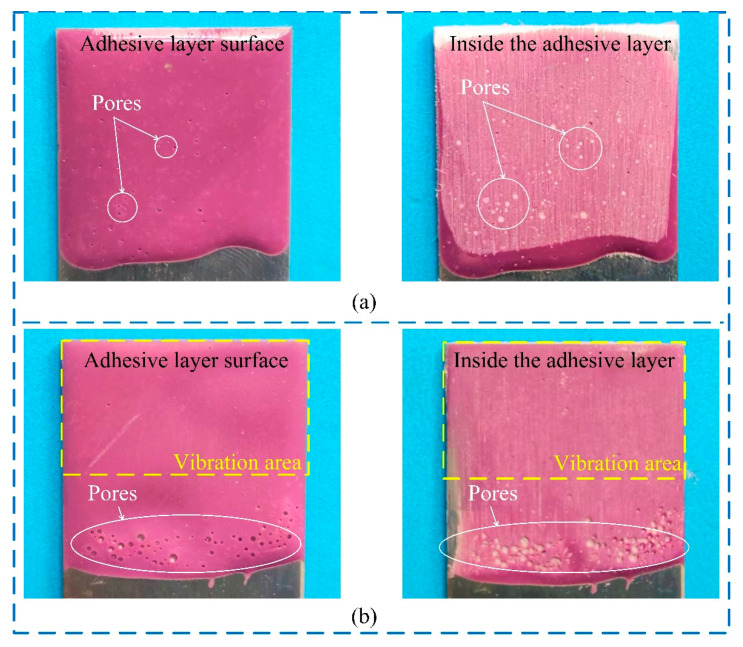
Pores in the cured adhesive (**a**) without and (**b**) with the ultrasonic vibration treatment.

In addition, the treatment has another advantage in reducing the porosity of the joint’s adhesive layer, as shown in Figure 10. The pores were a result of air trapped in the mixing process of the liquid adhesive and were hard to remove, as shown in Figure 10a. It was found that the bubbles were distributed in the adhesive away from the vibration area after the ultrasonic vibration treatment, as shown in Figure 10b. The mechanism was successfully used to reduce the porosity of the joints, as shown in Figure 7b. The pores in adhesive joints were found to have a negative influence on crack initiation in the adhesive layer and shear strength [33].

## 4. Conclusions

In this study, ultrasonic vibration was applied to join sandblasted aluminum alloy plates. The shear strength test and a hydrothermal aging process were used to evaluate the improvement of ultrasonic vibration on the bonding performance of aluminum alloy joints. In addition, an ultrasonic cavitation experiment was conducted to explain further the effect of ultrasonic vibration on the bonding interfaces. The conclusions are as follows.

(1) Compared with the joints without sandblasting treatment, the shear strength increased by 131.9% for the joints with the treatment, which reached 30.44 MPa. As to the joints with the sandblasting and ultrasonic vibration treatments, the shear strength increased to 33.33 MPa.
(2) After hydrothermal aging, the shear strength of the joints with sandblasting treatment decreased by 49%. It was 14% for the joints with the sandblasting and ultrasonic vibration treatment.
(3) With ultrasonic vibration treatment, the adhesive was in good contact with the micro-grooves on the aluminum alloy plate. It enhanced the joint shear strength. Moreover, the compact interfaces protected the aluminum alloy from corrosion.
(4) Many micro-jets produced by the cavitation effect were found in the adhesive by the aluminum foil erosion method. The pits on the aluminum foil surface showed that micro-jets have intensive impacts on the bonding interface. After the ultrasonic treatment, bubbles were gathered in the adhesive away from the vibration area, which contributed to a decrease in the adhesive porosity of joints.

## Figures and Tables

**Table 1:** Chemical composition of 7075 aluminum alloy plate.

Element	Al	Zn	Mg	Cu	Fe
Mass fraction/%	92.01	5.16	2.67	1.52	≤0.5

**Table 2:** Cured Adhesive Properties.

Property	LOCTITE EA 9309.3NA
Tension strength	32.2 MPa
Poisson’s ratio	0.36
Glass transition temperature (cured at 82 °C)	81 °C
Compressive strength	51.7 MPa

**Table 3:** Hydrothermal aging conditions.

Liquid	Temperature	Time
5% NaCl solution	80 °C	2 weeks

**Table 4:** Experimental plan.

Group	Sandblasting	Ultrasonic Vibration	Hydrothermal Aging
A	Without	Without	Without
B	With	Without	Without
C	With	Without	With
D	With	With	Without
E	With	With	With

**Table 5:** Factors and levels of the orthogonal experiment.

Levels	Factors		
	Gap Height (mm)	Moving Speed (mm/s)	Vibration Amplitude (μm)
1	0.5	2	16
2	1	4	20
3	1.5	6	24
4	2	8	28

**Table 6:** Orthogonal experimental schemes and results.

Scheme	Gap Height (mm)	Moving Speed (mm/s)	Vibration Amplitude (μm)	Average Shear Strength (MPa)
1	0.5	2	16	28.34 (1.61)
2	0.5	4	20	29.94 (0.71)
3	0.5	6	24	30.39 (0.44)
4	0.5	8	28	26.53 (0.89)
5	1	2	20	31.43 (0.77)
6	1	4	16	32.51 (0.54)
7	1	6	28	30.96 (0.21)
8	1	8	24	28.83 (1.03)
9	1.5	2	24	32.56 (0.54)
10	1.5	4	28	32.47 (0.81)
11	1.5	6	16	30.25 (1.04)
12	1.5	8	20	29.72 (0.73)
13	2	2	28	31.89 (0.58)
14	2	4	24	31.93 (0.64)
15	2	6	20	31.32 (0.76)
16	2	8	16	29.24 (1.15)

**Table 7:** Mean response (unit: MPa).

Level	Gap Height	Moving Speed	Vibration Amplitude
1	28.80	31.05	30.08
2	30.93	31.71	30.60
3	31.25	30.73	30.93
4	31.09	28.58	30.46
Delta	2.45	3.13	0.84
Rank	2	1	3

**Table 8:** Average shear strength and standard deviation of joints.

Group	Average Shear Strength (MPa)
A	14.42 (1.49)
B	30.44 (0.51)
C	15.54 (2.78)
D	33.33 (0.45)
E	28.66 (0.61)

## Data Availability

Data are contained within the article.
